# Virulence factors of *Phocoenobacter atlanticus* subspecies *atlanticus*: in search of vaccine targets

**DOI:** 10.3389/fmicb.2026.1793695

**Published:** 2026-05-18

**Authors:** Rebecca Marie Ellul, Hara Tselepidaki, Håkon Dahle, Håvard Skaar, Cyril Frantzen, Gyri Teien Haugland, Anita Rønneseth

**Affiliations:** 1 Department of Biological Sciences, University of Bergen, Bergen, Norway; 2 Institute of Marine Biology, Biotechnology and Aquaculture (IMBBC), Hellenic Center for Marine Research (HCMR), Heraklion, Greece; 3 STIM/ACD Pharma AS, Oslo, Norway

**Keywords:** Atlantic salmon, pasteurellosis, pathogenicity, *Phocoenobacter atlanticus* subsp. *atlanticus*, vaccine, virulence factors

## Abstract

Pasteurellosis caused by *Phocoenobacter atlanticus* subsp. *atlanticus* has a serious effect on Atlantic salmon welfare and the Norwegian aquaculture industry. Understanding the mechanisms underlying bacterial virulence through bioinformatic and functional analyses is an important contribution to vaccine development and prevention of disease. The genome of a clinical isolate of *Ph. atlanticus* subsp. *atlanticus* was sequenced and through *in silico* analysis 16 proteins were predicted to be adhesins, which are known virulence factors and may serve as potential vaccine targets. Expression levels of two such adhesins, a maltoporin-like protein and a 47 kDa outer membrane protein (Omp47), were studied following broth culture of the bacteria at 15°C, 20°C and 30°C and found to increase significantly over time during exponential growth phase from 20°C and higher (Omp47) and at 30°C (maltoporin). This could reflect field observations indicating higher frequency of pasteurellosis outbreaks following thermal delousing treatments. Further research on these target proteins may provide an important basis for development of vaccines against pasteurellosis in Atlantic salmon.

## 1 Introduction

The bacterium *Phocoenobacter atlanticus* subspecies *atlanticus*, previously named *Pasteurella atlantica* genomovar *salmonicida* (Nilsen et al., 2025b), causes pasteurellosis, which severely affects farmed Atlantic salmon (Nilsen et al., 2025a). As (Gulla et al., 2023). Clarify, the colloquial name for this disease has previously been wrongly associated with disease caused by *Photobacterium damselae* subsp. *piscicida* (family Vibrionaceae). In this study, the correct use of the term is applied to mean the disease caused by a *bona fide* member of the family Pasteurellaceae.

While isolated outbreaks of pasteurellosis have been recorded since 1989 (Valheim et al., 2000), steady outbreaks have been occurring since 2018 with gradually increasing frequency over the last 7 years (Nilsen et al., 2026). The disease affects fish from 2 kg up to harvest size (5 kg) and has been linked to stress caused by handling episodes and non-medicinal delousing treatments in the field. Gross pathology typically shows inflammation of the cardiac cavity, pseudobranchs and peritoneal cavity, along with lesions in skeletal muscle and at the basis of the fins. While exophthalmia has been recorded in early outbreaks, coining the name “varracalbmi” (Sami for “blood-eye”) (Valheim et al., 2000), and in some of the outbreaks since 2018, this is not always present. As a result of its severity, the disease poses a major challenge for Norwegian aquaculture due to reduced welfare and filet grading at harvest.

It has been suggested that *Ph. atlanticus* subsp. *atlanticus* should be considered as an opportunistic and not an obligate pathogen, since the bacterium has also been isolated alongside other bacterial and viral pathogens (Nilsen et al., 2025a, 2026; Stige et al., 2025). Work by Sandlund et al., 2021 highlighted the opportunistic nature of the pathogen, where under laboratory conditions, the study was not able to recreate disease in Atlantic salmon following a cohabitation challenge with lumpfish using both *Ph. atlanticus* subsp. *atlanticus* and *Phocoenobacter atlanticus* subspecies *cyclopteri*. Conversely, both subspecies were strongly pathogenic toward lumpfish.

The disease is not notifiable in Norway to date, however autogenous vaccines are currently in use, reflecting the impact this disease has on the health and welfare of the salmon (Nilsen et al., 2026). Although antibacterial treatment protocol has been established for *Ph. atlanticus* subsp. *cyclopteri* (Kverme et al., 2022) no significant commercial treatment regimens or prophylactic measures are available for *Ph. atlanticus* subsp. *atlanticus*. Characterization of the bacteria and identification of virulence factors and immunogenic protective antigens involved in pathogenicity seems to be the way forward to mitigate this disease in Atlantic salmon.

The first step in the bacterial infection process of a host is interaction with the host cell surface for attachment. Invasion of the cells and colonizing of tissues leads to infection and disease. Within the framework of reverse vaccinology, candidate virulence factors must be inherently immunogenic, eliciting a targeted antibody response in the host. Other major factors to be considered are subcellular localization, number of transmembrane helices and probability of adhesion functionality (Ong et al., 2020). For that reason, virulence factors that are released extracellularly (exotoxins), or that are membrane-bound on the bacterial surface are favored given the accessibility to host antibodies. Size and complexity of the protein may also influence immunogenicity (Casadevall and Pirofski, 2001).

Adhesins are a class of outer membrane virulence factors involved in attachment of the bacterium to the host, biofilm formation and evasion of host immune responses (Klemm and Schembri, 2000). They can be fimbrial (pili/flagella) or non-fimbrial (porins, transporters, proteases, nucleases). Following host attachment, adhesins can facilitate the transfer of toxins into the host leading to colonization of the host tissues which in turn triggers the host's innate immune response. The development of vaccines that can hinder the adhesion process would be advantageous to prevent pasteurellosis. A number of studies have identified immunogenic adhesins as vaccine targets which conferred protection from disease to a variety of fish species following challenge with *Aeromonas hydrophila* (Thirumalaikumar et al., 2022; Xu et al., 2024), *Edwardsiella tarda* (Jin et al., 2021), and *Vibrio harveyi* (Zhu et al., 2019). An adhesin, related to the adhesion protein Hia in *Heamophilus influenzae*, was also predicted by our group to be a virulence factor in *Ph. atlanticus* subsp. *cyclopteri*. Expression of the adhesin was upregulated both during cultivation of *Ph. atlanticus* subsp. *cyclopteri* in broth and following exposure to lumpfish head kidney leucocytes (Ellul et al., 2021).

Research on virulence mechanisms of *Ph. atlanticus* subsp. *atlanticus* is still in its infancy, limiting progress on vaccine development. The aim of this work was therefore to identify and investigate the virulence factors present in the *Ph. atlanticus* subsp. *atlanticus* proteome and predict via *in silico* and *in vitro* analyses potential antigens that can be used for vaccine development against pasteurellosis in Atlantic salmon.

## 2 Materials and methods

### 2.1 Bacterial culturing and isolation of genomic DNA

The four *Ph. atlanticus* subsp. *atlanticus* isolates (Paa-1-UiB-2019—Paa-4-UiB-2019) mentioned in this study were collected from commercial farms in Vestland county in Norway in 2019 from Atlantic salmon diagnosed with pasteurellosis. Paa-1-UiB-2019 was selected for DNA isolation and subsequent bioinformatic and gene expression analyses. Briefly, bacteria were grown in tryptic soy broth (TSB) (Becton Dickinson, Sparks, MD, USA) supplemented with 1.5% NaCl and 10% fetal bovine serum, Australian origin, (Gibco, Waltham, MA, USA) at 20°C with shaking (200 rpm).

Total genomic DNA was isolated using the DNeasy Blood and Tissue Kit (Qiagen, Hilden, Germany) following manufacturer instructions. Briefly, 3 mL of a culture of *Ph. atlanticus* subsp. *atlanticus* at late exponential growth phase containing a maximum of 2 x 10^9^ cells was centrifuged at 2,500 *g* for 15 min and the pellet was resuspended in 180 μL of ATL buffer. Proteinase K (20 μL) was then added, and the bacteria were incubated at 56°C in a rotating heat block for 1 h. AL buffer (200 μL) and 96% ethanol (200 μL) were added, vortexing well between additions. The mixture was then loaded onto DNeasy Mini Spin columns and washed with the appropriate buffers (AW1 and AW2) prior to elution using buffer AE. The eluted DNA was then measured for concentration and purity using NanoDrop2000 (Thermo Scientific) before storing at −20°C until whole genome sequencing.

### 2.2 Genomic sequencing, assembly and annotation.

Extracted DNA was sequenced using an Illumina platform. Illumina libraries were made using the Nextera DNA Flex Sample Prep kit (Illumina, San Diego, CA, USA) according to the manufacturer instructions and sequenced with Illumina MiSeq (Illumina, San Diego, CA, USA) using V3 chemistry. Raw sequences were adapter trimmed, quality filtered (Q > 20), *de novo* assembled using SPAdes (version 3.14.1) (Nurk et al., 2013). Contigs shorter than 1,000 bp or with < 5 times coverage were removed from each assembly prior to gene prediction. Genes were predicted, translated and annotated using Prokka (version 1.14.6) (Seemann, 2014).

This whole genome project has been deposited at GenBank under the Accession Number JBTTGU000000000.

### 2.3 Bacterial growth curves

Paa-1-UiB-2019 was cultured as described in Section 2.1 at 15°C, 20°C and 30°C to investigate the impact of temperature on growth and outer membrane protein development. Growth curves were compiled by measuring bacterial cell numbers using a cell counter (CASYModel TT (Innovatis) and CASY worX V1.26.

### 2.4 Visualization of major bacterial proteins by SDS-PAGE and staining

#### 2.4.1 Whole bacteria profiles and silver staining

Bacteria (Paa-1-UiB-2019—Paa-4-UiB-2019) were harvested in late exponential growth phase and centrifuged as described in Section 2.1. Cell numbers were adjusted by dilution in PBS to a final concentration of 8 x 10^8^ cells in 200 μL, giving a concentration of 4 x 10^8^ cells when diluted in sample buffer. When 10 μL of bacterial samples were supplied to the wells this gave a concentration of 1 x 10^7^ cells per well.

Protein profiles from whole bacteria were obtained by SDS-PAGE (12% acrylamide) according to the method of (Laemmli, 1970) with minor modifications as follows. Electrophoresis was performed using a Mini Protean Tetra Cell (Bio-Rad). The bacterial samples were mixed in a ratio of 1:1 in SDS Reducing Buffer supplied with β-mercaptoethanol (1:20) and heat-treated (98°C for 5 min) before 10 μL of the samples were supplied to the wells. 5 μL of unstained low range SDS-PAGE standard (Bio-Rad) diluted 1:20 in the SDS Reducing Buffer, treated equally as the bacterial samples, were used as ladder. The samples were electrophoresed at 190 V for 50 min, followed by fixation (62.5% methanol, 12.5% each of acetic acid, Fixative Enhancer Concentrate (Bio-Rad), and distilled water) and stained using Silver Stain Plus kit (Bio-Rad) based on the method of (Gottlieb and Chavko, 1987).

#### 2.4.2 Bacterial outer membrane and Coomassie staining

Bacteria (Paa-1-UiB-2019) were cultured as described in Section 2.3 in 500 mL volumes per growth phase tested and harvested at early, middle, and late exponential growth phase. Upon harvesting and centrifugation, bacterial pellets were resuspended in 1 mL 10 mM HEPES/5mM EDTA buffer and stored at−20°C until further processing. The samples were thawed on ice and sonicated at 40 μA for 30 min on ice, then centrifuged at 2,000 *g* for 5 min at 4°C. The supernatant was then collected and centrifuged at 12,000 *g* for 10 min at 4°C. The pellets were then washed three times using 1 mL HEPES/EDTA buffer. On the third wash, the samples were centrifuged at 27,000 *g* for 15 min at 4°C. The resulting pellets was dissolved in 170 μL 1 mM HEPES buffer supplemented with 25% Triton-X and incubated at room temperature for 20 min. Samples were then centrifuged at 12,000 *g* for 15 min at 4°C. The pellets were then washed twice with MilliQ water and subsequently resuspended in 1 mL MilliQ water and stored at−80°C for 24 h prior to freeze drying.

Outer membrane protein profiles were obtained by SDS-PAGE (12% acrylamide) according to the method of (Laemmli, 1970) with minor modifications as follows. Electrophoresis was performed using a Mini Protean Tetra Cell (Bio-Rad). The samples were reconstituted in SDS Reducing buffer supplemented in a 1:50 ratio with 2.5 M DTT and heat treated (98°C for 5 min) before 10 μL samples were supplied to gels. The same SDS-PAGE standard was used as described in Section 2.4.1. The samples were electrophoresed at 190 V for 50 min then stained overnight using Coomassie Brilliant Blue. After staining, the gels were destained prior to imaging (GelDoc Go Imaging System (Bio-Rad) and Image Lab Touch Software V 3.0.0.07).

### 2.5 In silico analyses of the predicted proteome

#### 2.5.1 Reverse vaccinology

The proteome of *Ph. atlanticus* subsp. *atlanticus* was screened against several databases to identify potential virulence factors. Firstly, the proteome was examined using PSORTb v.3.0.2 (Yu et al., 2010) to determine the subcellular localization of proteins, using a localization threshold of >7.5. Proteins predicted to be localized extracellularly or on the outer membrane were then screened against the virulence factor database VICTORS (Sayers et al., 2019) to identify potential virulence using an e-value of 1 x 10^−10^ to avoid false positives. Any hypothetical proteins were identified through BLASTp (Camacho et al., 2009) analysis against the non-redundant clustered protein sequences database. Comparisons of selected proteins were also made to publicly available genomes of *Ph. atlanticus* in this database, based on data compiled by (Gulla et al. 2023). Presence of signal peptides were predicted by SignalP (Almagro Armenteros et al., 2019) with a threshold of >90% to identify any known types of signal peptides and the location of their cleavage sites. Adhesion probability was tested using SPAAN (Sachdeva et al., 2005) with a threshold of 50% and the presence of transmembrane helices was detected using TMHMM2 (Krogh et al., 2001) (score ≤ 1).

Genomic islands, which are clusters of genes of probable horizontal origin, were identified using IslandViewer4 (Bertelli et al., 2017), while PHASTER (Arndt et al., 2016) was used to detect and classify inducible prophages.

Several tools were then used to identify properties related to immunogenicity. VaxiJen2 (Doytchinova and Flower, 2007) with a threshold of 0.4, was used to identify protective antigen probability, ToxinPred2 (Sharma et al., 2022) predicted toxin potential of the proteins, while BepiPred 3.0 (Clifford et al., 2022) was used to predict the most probable linear B cell epitope regions on each protein, with a threshold of 0.1512 and confidence set to return the top 20% of epitopes.

The interaction between the predicted B cell epitopes and MHC II complex of Atlantic salmon was further investigated through homology modeling. The α (Q5ZQM4_SALSA) and β (Q95IS0_SALSA) chains of Atlantic salmon MHC II, retrieved from the SWISS-MODEL repository, were docked using the ClusPro V2.0 server (Kozakov et al., 2017). The quality of the produced MHC II model was assessed using the MolProbity web server (Williams et al., 2017). The CABS-dock webserver (Kurcinski et al., 2015) was used to model and predict the binding sites of the predicted epitopes to the MHC II complex by considering peptide flexibility and small fluctuations of the receptor backbone. Subsequently, the webserver PRODIGY (Xue et al., 2016) was employed to predict the binding affinities and dissociation constants of the resulting protein–peptide complexes. All parameters were set to default settings.

#### 2.5.2 Structural visualization

To enable structural visualization and epitope mapping, three-dimensional (3D) models of these proteins were generated using two online homology modeling platforms: AlphaFold (Fleming et al., 2025) and SWISS-MODEL (Waterhouse et al., 2018). For each protein, the model exhibiting a higher percentage of residues within the favored regions of the Ramachandran plot was selected. A threshold of >98% of residues in the favored regions was established as the criterion for a high-quality, near-native protein conformation.

Subsequently, the GalaxyRefine (Heo et al., 2013) web server was employed to enhance the stereochemical quality of the predicted 3D protein structures. The refined models were evaluated based on the statistical parameters provided by GalaxyRefine, and the most optimal models were selected according to the best combination of quality metrics. The stereochemical properties of the selected structures were then re-assessed using Procheck (Laskowski et al., 2012), which generated updated Ramachandran plots.

The predicted B cell epitopes were then visualized and highlighted on the three-dimensional structures using PyMOL V3.0 (The PyMOL Molecular Graphics System, Version 3.0 Schrödinger, LLC.). In addition, the transmembrane topology and subcellular localization of each protein were annotated using DeepTMHMM (Hallgren et al., 2022). Based on these data, distinct structural regions were color-coded for clarity: transmembrane regions were represented in magenta, periplasmic regions in blue, and outer membrane segments in green. Signal peptides were depicted in gray, while the predicted epitopes were highlighted in red.

### 2.6 Analysis of gene expression during bacterial exponential growth

#### 2.6.1 Processing bacteria for qPCR analysis

Cultures of *Ph. atlanticus* subsp. *atlanticus* used for gene expression analysis during bacterial cell proliferation in growth medium were set up at 15°C, 20°C and 30°C, and incubated as described in Section 2.1. Bacteria were harvested at early, middle and late exponential growth phase for each of the temperatures. Samples were taken in triplicate and adjusted 1 x 10^9^ bacterial cells mL^−1^, based on counts measured at harvest using a cell counter (CASY Model TT (Innovatis) and CASY worX version 1.26) followed by centrifugation at 2500 *g* (Beckman Coulter Allegra X-15R) for 10 min at 4°C. The growth medium was then discarded, and the pellets stored immediately at−80°C. Total RNA was then extracted using the Bacterial RNA Kit (E.Z.N.A., Norcross, GA, USA) according to manufacturer instructions. The RNA was then DNase-treated (Sigma-Aldrich, Saint-Louis, MO, USA), converted to cDNA using qScript cDNA Synthesis Kit (Quantabio, Beverly, MA, USA), and stored at-−20°C. For qPCR, 6.25 ng μL^−1^ of cDNA was used as the template for both the target and reference genes.

#### 2.6.2 qPCR

Each qPCR reaction contained a volume of 10 μL and consisted of 5 μL 2 × SYBR Green JumpStart Taq Ready Mix, 0.4 μL each of the forward and reverse primers (10 μM final working concentration, Table 1), 0.2 μL of RNase and DNase free water (Sigma), and 4 μL of cDNA. A C1000 Touch thermal cycler with CFX96 Real-Time System (Bio-Rad) was used for qPCR, with the following cycle conditions: 94°C for 5 min followed by 40 cycles of 94°C for 15 s and 60°C for 1 min. Melting curve analyses were performed after each run (60 to 92°C at a rate of 1°C/5 s). Three parallel reactions were performed for each sample, and negative controls excluding cDNA (NTC) and cDNA reactions without reverse transcriptase (NRT) were included for all master mixes.

**Table 1:** qPCR primer details for the two reference and two target genes.

Gene	Primer name	Sequence 5′−3′	Primer length (bp)
*gyrA*	gyrA _F	ATGAGCGTATCACAGCGATCTTACC	25
	gyrA_R	CTTGCGGTTGCCATCACTACGA	22
*rpoD*	rpoD_F	CCACAACTGCTTCTGCGATAGGAC	24
	rpoD_R	CGGCTCCATCTGGTGCATCAAC	22
* < mpor>*	mPorin_F	TCAACGCCACGATGTCCACATG	22
	mPorin_R	CAAGTACAGCACCGCCACTCTC	22
* < omp47>*	porin_F	AATGCAATTCAAGCTGAGGCTGAGT	25
	porin_R	GCAAGACCAACATTCCAACCATAGC	25

<*mPor*> refers to gene coding for the maltoporin-like protein, < *omp47*> refers to the gene coding for the 47 kDa outer membrane protein.

#### 2.6.3 Primer design and validation

Genes considered for this work were based on results from the above analyses of potential vaccine targets. The reference genes were *rpoD* and *gyrA*. Two candidate genes (coding for a 47 kDa outer membrane protein (Omp47) and a maltoporin-like protein (maltoporin)) were shortlisted as potential target genes, based on impact on cell adhesion.

qPCR assays were designed using the software Primer Premiere (v. 6.24). The five highest rated assays for each target sequence were then chosen for testing. The length of the amplicons was kept between 100–250 bp for optimal amplification efficiency. The specificity of the primers was confirmed by qPCR (20 ng cDNA used in each qPCR reaction) and product size observed by electrophoresis on 2% agarose gels. All qPCR assays produced single amplification products as expected (data not shown). The best assay for each target gene based on Cq value, non-template controls, melting curves and results of electrophoresis was then chosen for further work. The resulting primers used for qPCR are listed in Table 1.

### 2.7 Statistical analysis

The fold change in gene expression was calculated by the ΔΔCq method using *gyrA* as reference gene and mean normalized expression was folded against the early time point. The results were analyzed using two-way ANOVA and the Holm-Sidak method for *post hoc* tests, and differences were considered significant when *p* < 0.05. All statistical analyses were carried out using SigmaPlot version 15.0 (Systat Software, San Jose, CA, USA) and reports are available as Supplementary Data 1 (maltoporin) and 2 (Omp47).

## 3 Results

### 3.1 Genomic elements

The genome of *Ph. atlanticus* subsp. *atlanticus* is 2,167,797 base pairs long with a GC content of 34.26% and 1,987 coding regions.

#### 3.1.1 Genomic islands and prophages

**Figure 1:**
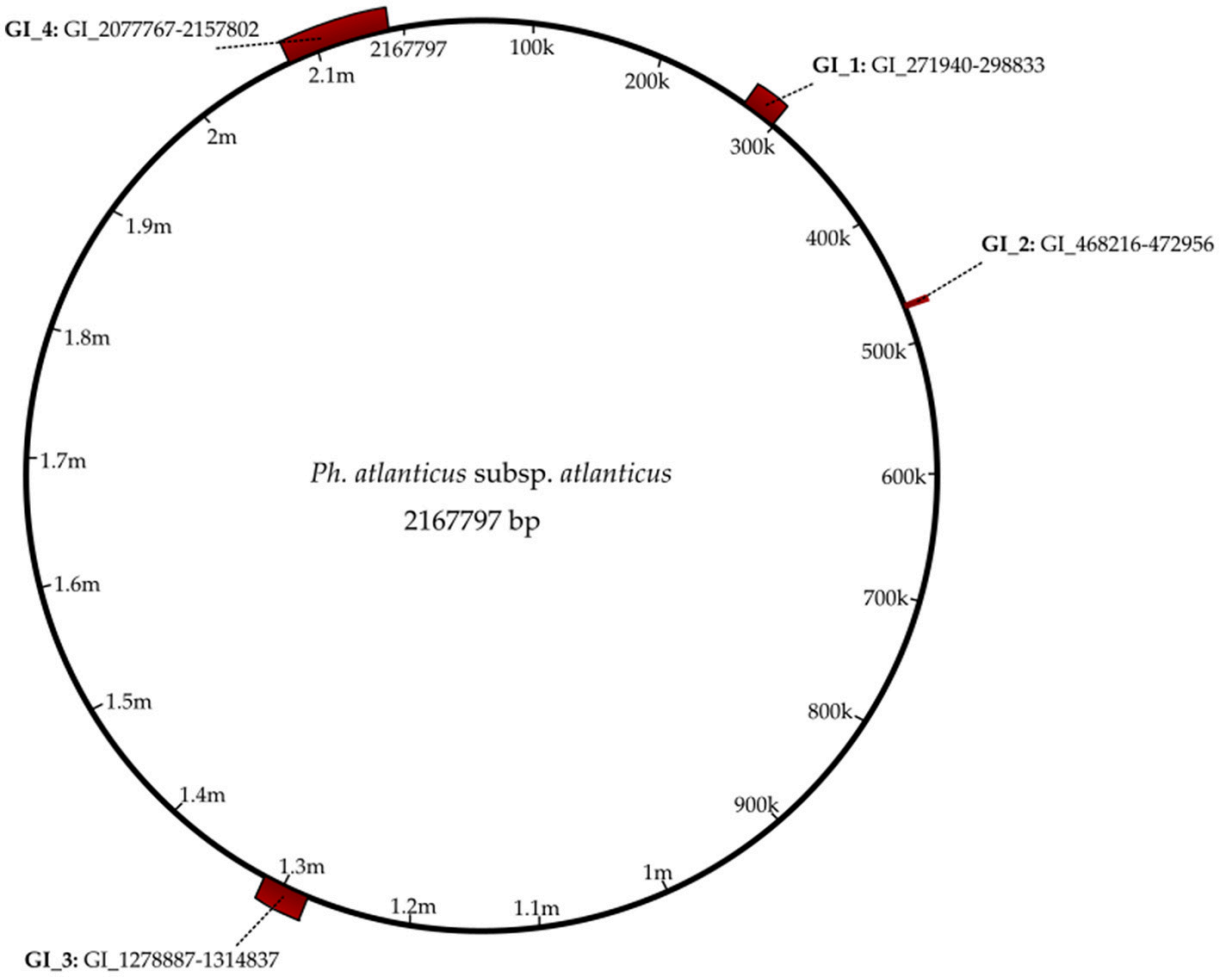
Genomic map of *Ph. atlanticus* subsp. *atlanticus* highlighting the four genomic islands present.

Four genomic islands (GI) were identified (Figure 1), with a GC content between 31.03 and 34.75% (Table 2). The majority of the 88 coding sequences encompassed by the genomic islands are as yet uncharacterized (hypothetical proteins) and constitute approximately 4.4% of the entire proteome. Toxin/antitoxin (TA) systems are present in GI_3 and GI_4. Coding sequences (CDS) of HigA and HigB, which are an antitoxin and a ribosome-dependent endonuclease, respectively, were present on both these islands, indicating that two copies of the system are present in the proteome. A gene coding for the toxin FitB, part of the FitAB TA system was also present on GI_4. Several genes coding for proteins forming part of insertion sequences (IS) were also present on GI_4.

**Table 2:** Features of the four genomic islands (GI) found in the *Ph. atlanticus* subsp. *atlanticus* genome.

#	Sequence location	Sequence length (bp)	GC%	CDS
GI_1	GI_271940-298833	26,893	32.99	9
GI_2	GI_468216-472956	4,740	31.03	7
GI_3	GI_1278887-1314837	35,950	34.75	31
GI_4	GI_2077767-2157802	80,035	34.14	41

Proteome analyses did not reveal the presence of inducible prophages, only one incomplete prophage, although a gene coding for Prophage integrase IntA is present on GI_3.

#### 3.1.2 Identification of virulence factors and vaccine candidates

The proteome consisted of 1,987 predicted coding sequences. Since the focus of this study was virulence factors that were surface proteins, protein sequences were first analyzed using PSORTb to identify subcellular localization and filter out presumed outer membrane and extracellular proteins. These amounted to 58 proteins, representing 3% of the total number of proteins. Among these proteins, 16 were predicted to be adhesins using SPAAN, and hence likely to be related to adhesion, and thus, virulence. These correspond to 0.8% of the proteome (Table 3).

**Table 3:** Overview of outer membrane and extracellular putative adhesins in the *Ph. atlanticus* subsp. *atlanticus* proteome.

#	CDS	PDB Header (molecule)	Localization (%)	Molecular weight (kDa)	Adhesin (%)
1	Hypothetical protein	Ribonuclease (Colicin E3)	O (100)	574.65	93.1
2	Hypothetical protein	Structural protein (WD40 domain protein)	O (95)	38.18	91.5
3	Toxin CdiA	membrane protein/chaperone (AopB)	E (96)	30.00	89.8
4	Hypothetical protein	Cell adhesion (23S rRNA pseudouridine synthase D, PilA)	E (96)	16.00	87.3
**5**	**Hypothetical protein**	**Biosynthetic protein (Periodic tryptophan protein 2)**	**O (95)**	**61.73**	**83.4**
6	Hypothetical protein	Hydrolase (Cell wall binding endopeptidase-related protein)	O (99)	15.54	83.2
**7**	**47 kDa outer membrane protein**	**Lipid transport (Long-chain fatty acid transport protein)**	**O (100)**	**45.43**	**82.3**
8	Secretory immunoglobulin A-binding protein EsiB	unknown function (SEL-1 repeat protein)	E (97)	23.98	81.9
**9**	**Maltoporin**	**Virus (Maltoporin)**	**O (100)**	**44.74**	**80.6**
10	Hypothetical protein	toxin (Diphtheria toxin)	O (100)	231.92	80.3
11	Hypothetical protein	Lipid binding protein (Lipoprotein)	O (99)	14.63	79.9
12	Hypothetical protein	Motor protein (Kinesin light chain 1, Torsin-1A)	O (99)	21.62	79.7
**13**	**Toxin and drug export protein A**	**Transport protein (CmeC)**	**O (100)**	**52.03**	**76.6**
14	Protein YceI	hydrolase (Esterase estA)	O (95)	121.36	75.4
15	Hypothetical protein	cell adhesion (filamentous hemagglutinin)	E (84)	238.61	74.9
**16**	**Hypothetical protein**	**Cell invasion (Putative type VI secretion system YD repeat-containing Rhs element Vgr protein)**	**E (95)**	**56.90**	**74.8**

O: outer membrane, E: extracellular. The four outer membrane and one extracellular putative virulence factors fitting in the 40-62 kDa size range are marked in bold.

**Figure 2:**
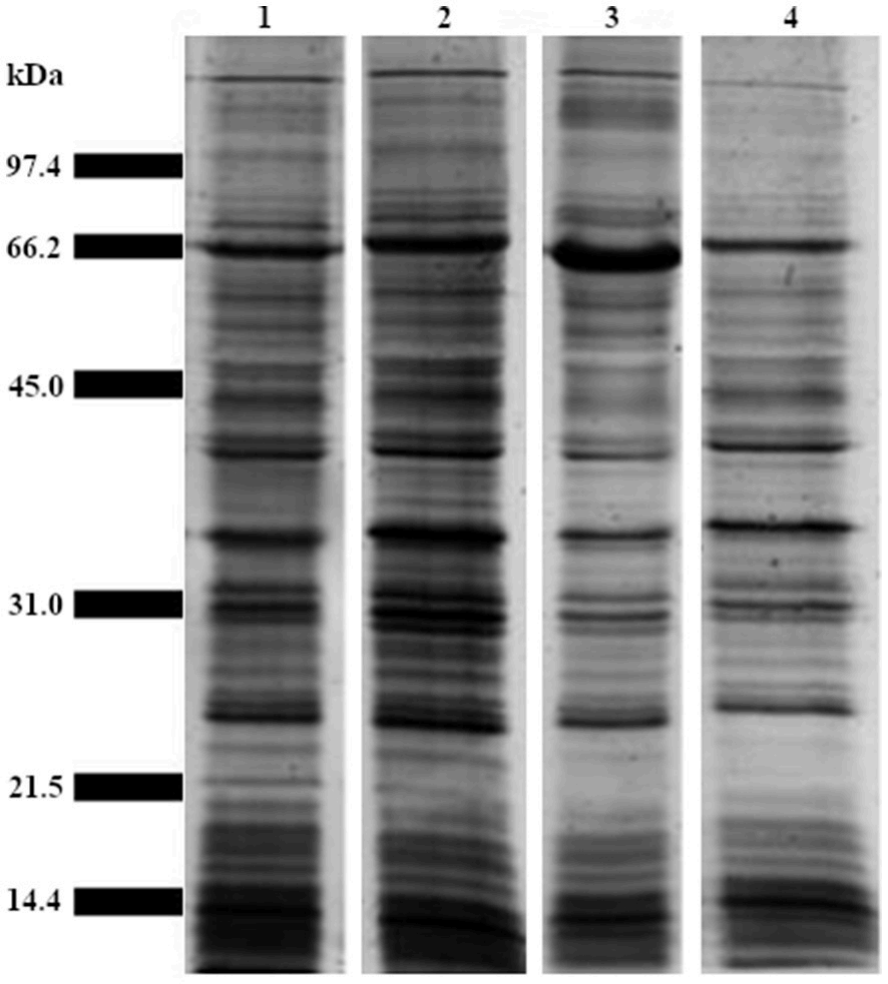
Silver stained SDS-PAGE gel showing protein profiles of four in-house *Ph. atlanticus* subsp. *atlanticus* isolates (Paa-1-UiB-2019—Paa-4-UiB-2019) marked 1–4, respectively. The profile for the isolate used in this study is shown in lane 1 (Paa-1-UiB-2019).

**Figure 3:**
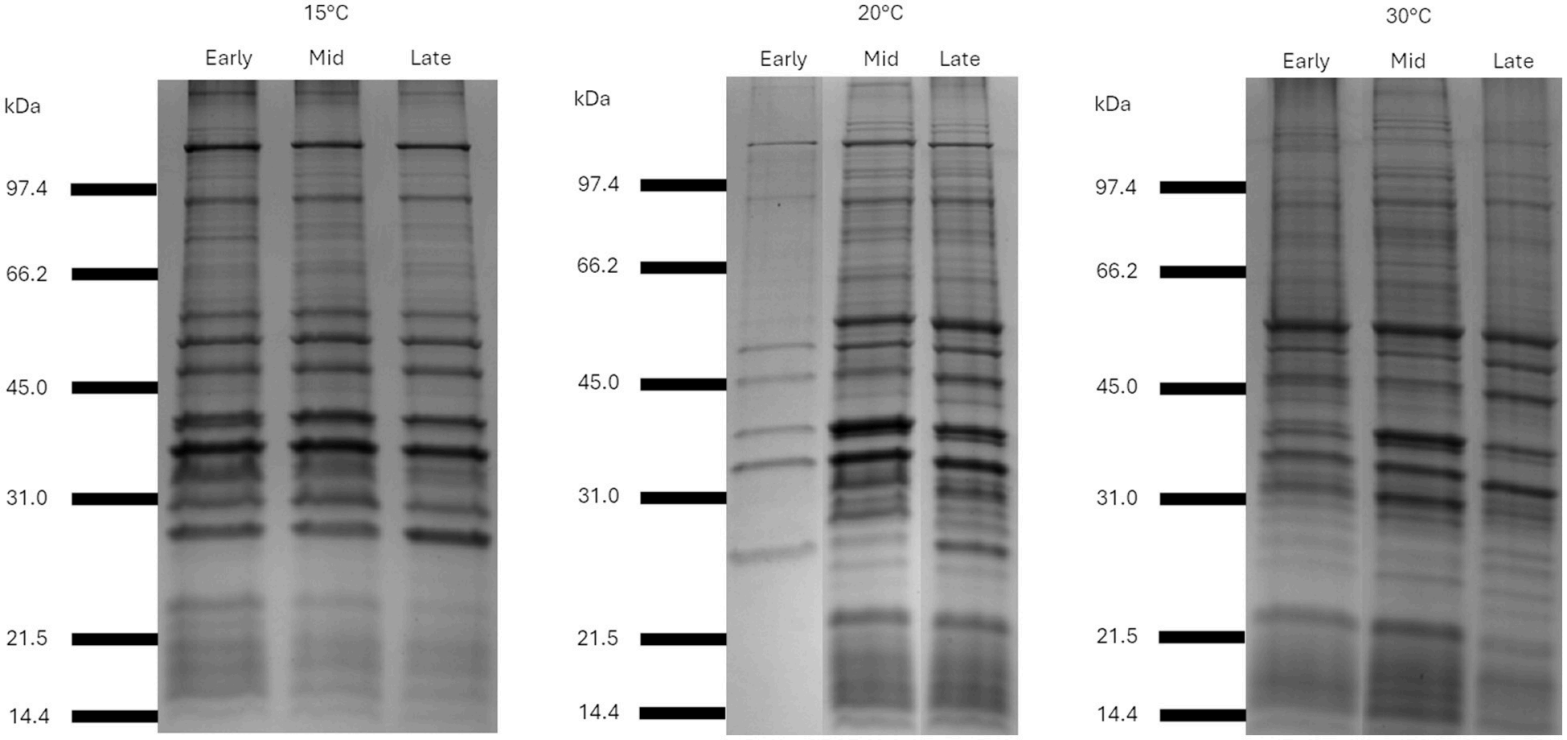
Coomassie Blue stained SDS-PAGE gels showing protein profiles of the outer membrane of Paa-1-UiB-2019 harvested at early, middle, and late exponential phase, at 15°C, 20°C, and 30°C.

Analysis of the whole bacteria protein profiles of the four *Ph. atlanticus* subsp. *atlanticus* isolates and the outer membrane protein (OMP) profiles indicated that proteins in the 40–66kDa molecular weight range could be of interest (Figures 2 and 3). Furthermore, these bands were present at all temperatures and growth phases analyzed. When applying this filter to the 16 predicted adhesins, five proteins were identified that fall in the 40-66 kDa range (Table 3, marked in bold).

Further analyses into identifying immunogenicity traits and vaccine-related properties were thus carried out on these five proteins (Table 4). All the proteins had only one or no transmembrane (TM) helices, an antigen probability higher than 55%, and only one (Toxin and drug export protein A) was predicted to be a toxin. Secretory signal peptides were present in three of the five adhesins, where following transport by the Sec translocon two would be cleaved by Signal Peptidase I (Maltoporin and 47 kDA outer membrane protein [Omp47]), while the other by Signal Peptidase II (Toxin and drug export protein A). Two of the adhesins, the maltoporin-like protein and Omp47 were chosen for further investigation based on their strong adhesin potential.

**Table 4:** Immunogenic properties of the predicted adhesins.

#	CDS	Signal peptides	TM helices	Antigen probability	Toxin Prediction	B cell epitopes present
5	Hypothetical protein	n/a	0	0.5551	Non-Toxin	Y
7	47 kDa outer membrane protein	SP Sec/SPI	1	0.7489	Non-Toxin	Y
9	Maltoporin	SP Sec/SPI	1	0.6822	Non-Toxin	Y
13	Toxin and drug export protein A	Lipo Sec/SPII	0	0.5769	Toxin	Y
16	Hypothetical protein	n/a	0	0.7063	Non-Toxin	Y

SP: Signal peptide, Lipo: lipoprotein.

**Figure 4:**
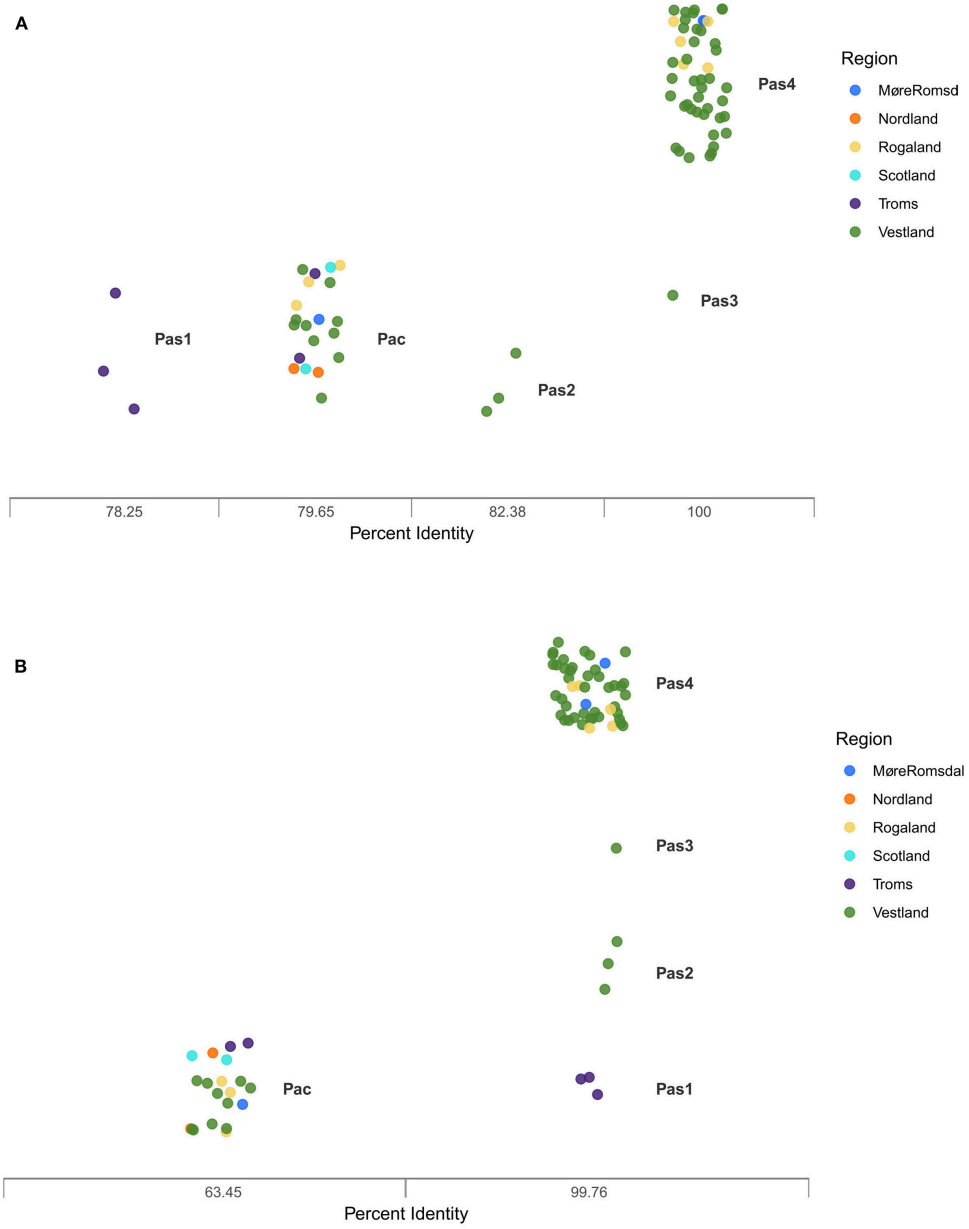
Results from BlastP comparison of maltoporin **(A)** and Omp47 **(B)** from Paa-1-UiB-2019 to publicly available genomes of *Ph. atlanticus*. Pas: *Ph. atlanticus* subsp. *atlanticus*. Pac: *Ph. atlanticus* subsp. *cyclopteri*. Pas1 (late 1990s), Pas2 (early 2000s), Pas3 (2012), Pas4 (2018-2020), Pac (1996-2018). Colored points refer to region of collection of isolates.

BlastP analysis of the two adhesins from Paa-1-UiB-2019 against the ClusteredNR database revealed that maltoporin and Omp47 are strongly identical to the same adhesins present in publicly available genomes of *Ph. atlanticus* subsp. *atlanticus* on Genbank (Figures 4A, B; Supplementary Table 1 and 2). In the case of maltoporin, it is 100% identical to the same protein found in *Ph. atlanticus* subsp. *atlanticus* isolates collected in 2013 (Pas3) and between 2018 and 2020 (Pas4), and gradually less identical to that found in isolates collected before 2013 (Pas1, Pas2) and in *Ph. atlanticus* subsp. *cyclopteri* isolates (Pac) (Figure 4A). Omp47 is 99.76% identical to the *Ph. atlanticus* subsp. *atlanticus* isolates irrespective of year of collection (Pas1-4), and only 63.45% to *Ph. atlanticus* subsp. *cyclopteri* isolates (Pac) (Figure 4B).

#### 3.1.3 B cell epitope docking to Atlantic salmon MHC class II and visualization of models

The highest-ranking predicted epitopes for the maltoporin-like protein and Omp47 were selected for protein-peptide docking to MHC II from Atlantic salmon. The model for the joined sub-units of MHC class II was favorable (96.1% residues in favored regions (goal >98%); 99.3% of all residues in allowed regions (goal >99.8%). The docking showed moderately strong binding affinities (−6.5 to−9.7 kcal mol^−1^) and dissociation constants (2.5 x 10^−6^ to 6.5 x 10^−8^ M) (Table 5).

**Table 5:** Predicted epitopes for maltoporin-like protein (maltoporin) and Omp47 and their binding affinities and dissociation constants to Atlantic salmon MHC II.

Protein	Epitope	Binding affinity (kcal mol-1)	Dissociation constant (M) at 10 °C
Omp47	*DGKLRTPRGNMDASNG*	−8.9	1.3 x 10^−7^
	GVLIPQKMDT	−7.2	2.9 x 10^−6^
	SMSPKPVLE	−8.8	1.7 x 10^−7^
	FVEVQKEAPM	−7	3.6 x 10^−6^
Maltoporin	*GGGQPLLQ*	−7.3	2.5 x 10^−6^
	SQKVDWESTS	−7.9	7.4 x 10^−7^
	ESGGAVLGAEKAD	−9.7	3.3 x 10^−8^
	AKDGAKFVDGAT	−9.3	6.5 x 10^−8^
	SGHSQGSRVD	−6.5	8.9 x 10^−6^
	QQKDGKK	−8	6.2 x 10^−7^

The italicized epitope was used for structure visualization using AlphaFold.

**Figure 5:**
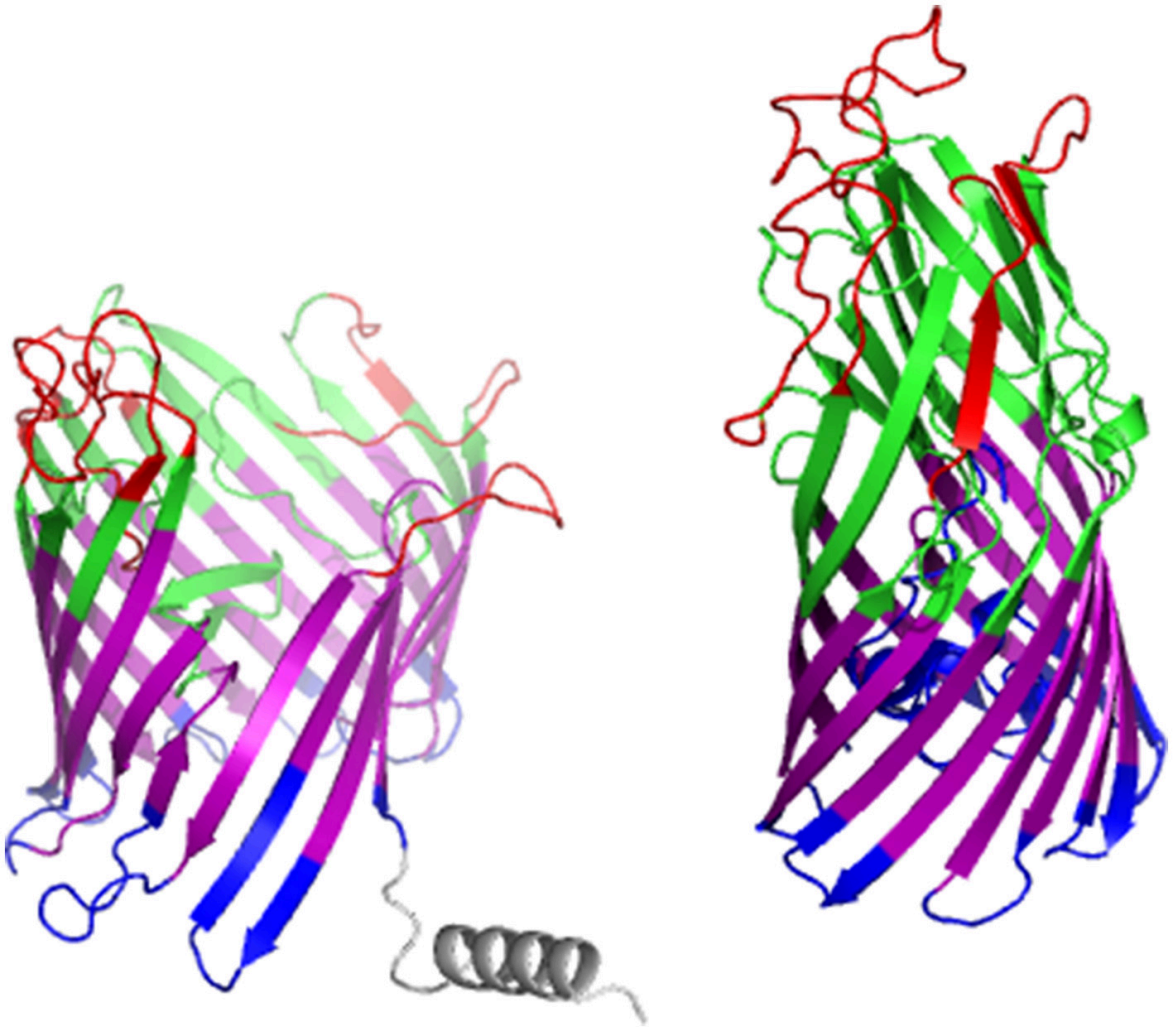
AlphaFold renditions of the two adhesins maltoporin and porin. Red: epitope, green: extracellular, magenta: outer membrane, blue: periplasm, gray: signal peptide.

Models built using AlphaFold visualize the predicted epitopes in relation to the predicted organization of the protein (Figure 5). The predicted epitopes lie in the extracellular domains of the proteins.

### 3.2 Gene expression of the major virulence factors at different growth phases

**Figure 6:**
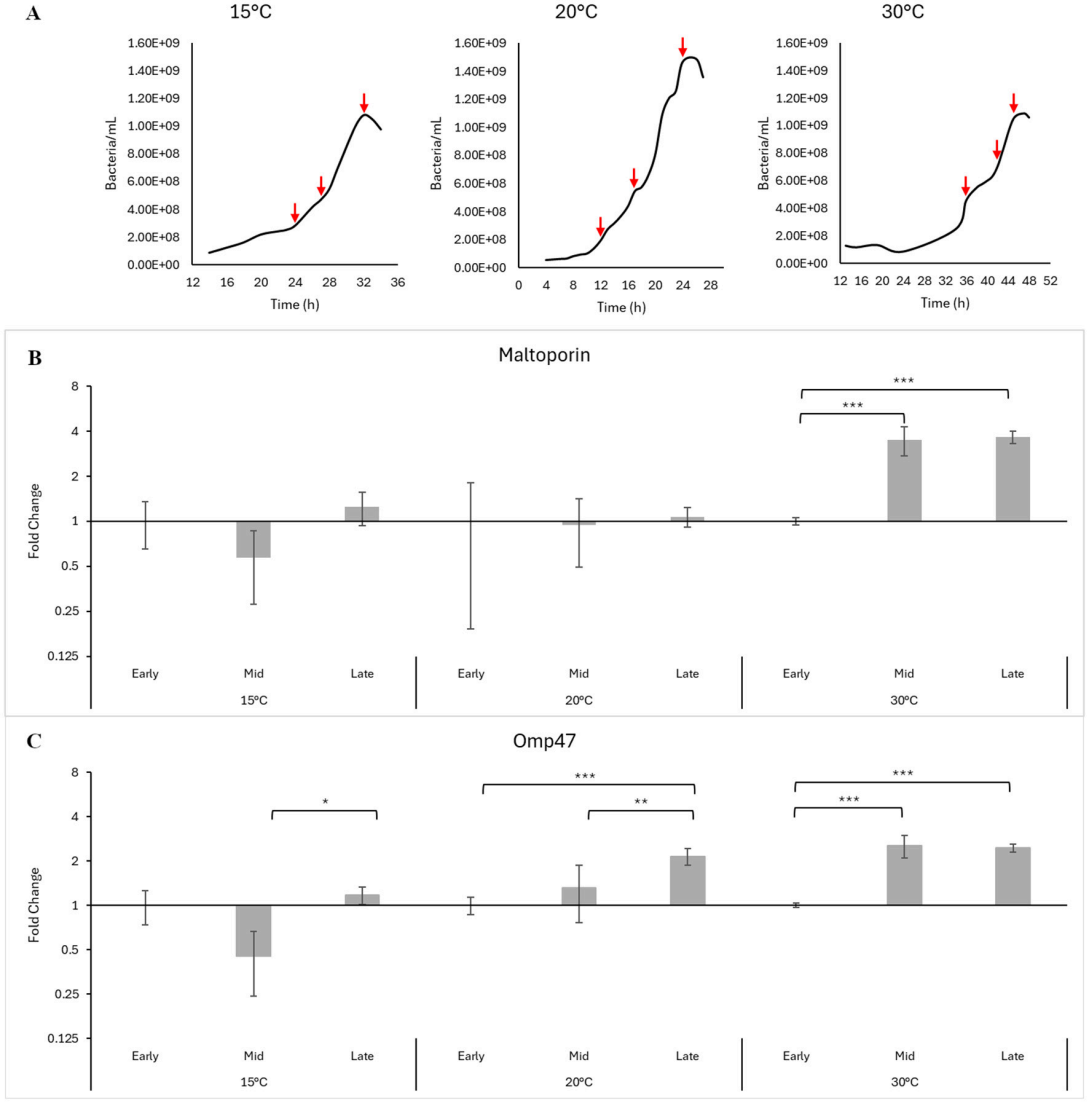
**(A)** Growth curves for Paa-1-UiB-2019 cultured at 15°C, 20°C, and 30°C. Red arrows indicate harvest points to coincide with early, middle, and late exponential growth phases. Gene expression of maltoporin **(B)** and Omp 47 **(C)** at different growth curve stages at 15°C, 20°C, and 30°C. Error bars show standard deviation. Asterisks indicate statistically significant differences, where *(*p* ≤ 0.05), ** (*p* ≤ 0.001), and *** (*p* ≤ 0.0001); *n* = 3.

The timepoints chosen for measurement of gene expression were based on growth curves established for the bacteria (Figure 6A), to coincide with the early, middle and late stages of the exponential growth phase. Of the reference genes tested, *gyrA* gave the most stable readings across all three temperatures investigated in this study and was thus used as the reference gene for statistical analysis (Supplementary Figure 1).

The expression of the two proteins seems to generally be affected by an increase in temperature (Figures 6B, C and Supplementary Table 3).

At 15°C and 20°C expression of the maltoporin gene was largely unchanged throughout the growth cycle (*p* > 0.05), while at 30°C a significant 3.5 and 3.7-fold increase could be observed during mid and late exponential phase, respectively, compared to the early exponential phase (*p* < 0.001) (Figure 6B). Furthermore, expression of this gene increases significantly (*p* < 0.001) from mid exponential phase onwards at 30°C (Supplementary Table 3).

At 15°C, gene expression for Omp47 was downregulated during the mid exponential phase compared to the early phase (*p* = 0.066) but is upregulated at the late exponential phase compared to the mid phase (*p* = 0.02). At 20°C expression increases significantly over time, to a two-fold increase by late exponential phase (*p* < 0.001), and at 30°C increases 2.5-fold over mid and late exponential phase (*p* < 0.001 for both), compared to early exponential phase (Figure 6C). Furthermore, expression of this gene seems to increase significantly (*p* < 0.001) from mid exponential phase onwards, from 20°C and higher, with the exception of the late exponential phases at 20°C and 30°C where expression is not significantly different (Supplementary Table 3).

## 4 Discussion

### 4.1 Genomic islands and search for intact and inducible prophages

Virulence genes are often found within genomic islands, which are typically abundant in pathogenic strains (Castillo et al., 2017). In the isolate of *Ph. atlanticus* subsp. *atlanticus* studied, only four genomic islands, and no intact prophages were identified in the genome. The limited presence possibly could be consistent with previous field observations indicating that the pathogenicity of *Ph. atlanticus* subsp. *atlanticus* is limited (Stige et al., 2025).

Proteins linked to toxin/antitoxin systems (TA) were present, namely HigA/HigB and FitAB. These are typically involved in anti-phage measures, biofilm formation, regulation of stress responses and pathogenicity (Wood and Wood, 2016). FitAB is typically involved in intracellular replication or cleavage of RNA which leads to impaired cell function. This is usually a response to phage infection or antibiotic persistence (Lee and Lee, 2016). There were also proteins linked to insertion sequences/elements, which are involved in sequestering, transmitting, mutating and activating genes (Siguier et al., 2015). We hypothesize that these results could explain the lack of intact or inducible prophages in the genome of *Ph. atlanticus* subsp. *atlanticus*, and in turn the reduced virulence. In contrast, as shown in (Ellul et al. 2021) the genome of *Ph. atlanticus* subsp. *cyclopteri* contained five intact prophages, of which four contained parts of, or entire genomic islands. This subspecies also has more mobile elements, and so more virulence genes. This could explain the higher virulence, at least to lumpfish (Sandlund et al., 2021) compared to *Ph. atlanticus* subsp. *atlanticus*. Analysis of other publicly available genomes of the bacteria will be necessary to test this hypothesis.

### 4.2 Virulence factors

Adhesins are a class of outer membrane proteins often used by pathogenic Gram-negative bacteria to facilitate attachment to biotic or abiotic surfaces, for colonization of hosts or for biofilm formation, respectively. They are thus a cornerstone of pathogenesis and survival for the pathogen, and are classed as virulence factors, making them suitable as potential vaccine targets for reverse vaccinology.

Of the 16 proteins classified as adhesins in this study, more than half were classified as hypothetical based on BlastP searches and required further analysis to determine identity. Hits from searches in VFDB were associated with high *e*-values (>1 x 10^−10^) indicating that hits may have been false positives. This was to be expected given the limited number of fish pathogenic bacteria recorded in these databases. In fact, all the hits were associated with human pathogenic bacteria. This highlights the need for dedicated databases for fish pathogens. Furthermore, given the great phylogenetic distance to other members of the Pasteurellaceae, the results were expected. Searches using other databases such as BlastP and Protein Database (PDB) provided some clarity, but still with limited success.

Through SDS-PAGE analysis, proteins belonging to the 40–66 kDa molecular weight range were considered for further analyses to investigate potential immunogenic roles due to the presence of the same bands in both the whole bacteria as well as the OMP samples of the bacteria. Of the 16 adhesins five fall in the size range, and two proteins classed as porins, a maltoporin-like protein (maltoporin) and a 47 kDa outer membrane protein (Omp47), were the point of focus of the current study based on their adhesin potential.

Maltoporin (or LamB porin), was first studied in *E. coli* as the receptor of the well-studied bacteriophage lambda (λ). It is involved in sugar translocation, specifically maltodextrin and maltose for the regulation of maltose in the bacterium (Chatterjee and Rothenberg, 2012). Furthermore, it has been identified as a major virulence factor of *Aeromonas veronii*, where it is involved in adhesion and its deletion resulted in greatly attenuated virulence of the mutated strain to zebrafish and mice (Yang et al., 2019). The structure in *E. coli* is made up of three identical B-barrels, whereas only one is present in *Ph. atlanticus* subsp. *atlanticus*. The incomplete structure could partly point to a strategy adopted to evade phage infection, as described by (Chatterjee and Rothenberg 2012), or incomplete horizontal gene transfer.

The Omp47 protein was identified as having a role in lipid transport according to PDB. Structurally, it is similar to FadL of *E. coli* which is involved in long-chain fatty acid (LCFAs, C < 12) transport for energy metabolism. Homologs of the protein are found in different bacteria, with roles ranging from degradation of xenobiotics as well as bacterial pathogenesis (Black and DiRusso, 2003). FadL is involved in host cell adhesion in non-typable *H. influenzae* (NTHi) to establish host colonization and infection. Following establishment, loss of the fadL gene seems to confer the pathogen with resistance to the bactericidal effects of arachidonic acid which is released by host cells following phagocytosis and following the action of bacterial phospholipases, suggesting antagonistic pleiotropy (Moleres et al., 2018).

Conversely, *E. coli* can metabolize arachidonic acid, through activation of genes involved in B-oxidation of LCFAs (*fadB*) by FadL and the enzyme FadD. Thus, the reduced concentration of the LCFA serves to suppress the host's local inflammatory response, resulting in the pathogen gaining an advantage in early colonization of the host (Black and DiRusso, 2003).

While BlastP results indicated Omp47 is a porin, FadL is not one as the periplasm side of the barrel is closed off by a hatch domain which limits direct connection with the extracellular environment (Waters and Eijkelkamp, 2024). Homologs of FadL typically show structural variation in the extracellular loops, which may be in response to evasion of phage infection, since FadL functions as a receptor of the T2 bacteriophage in *E. coli* (Black, 1988). The predicted structure of the Omp47 from the current study reflects the reported structures, and our analysis shows that the extracellular loops may be B cell receptor epitopes, although further work is required to better elucidate the definite structure of these proteins.

BlastP searches of the two adhesins may shed some light on the evolutionary development of maltoporin and Omp47. Interestingly, the gene encoding the maltoporin-like protein was only found in genomes collected between 2012 and 2020, while the gene encoding the Omp47 is present in all the available *Ph. atlanticus* subsp. *atlanticus* genomes ranging from 1991 until 2020. Neither gene is present at the same identity level in the *Ph. atlanticus* subsp. *cyclopteri* genomes available (79.65% for maltoporin and 63.45% for Omp47). Percent identities to *Ph. uteri* were even lower (not shown), indicating that the proteins may have undergone significant mutation over time and across host species. One could also speculate on the potential link between evolutionary age of the subspecies and pathogenicity. The salmon subspecies has been isolated since the late 1980s with a characteristically low virulence, while the lumpfish subspecies was first recorded in 2012 (Alarcón et al., 2016) and is substantially more pathogenic. It is possible that the pathogen evolved to stronger virulence as it expanded its host range, although this will need in depth investigation.

Predictive protein-peptide docking analysis of the respective epitopes to Atlantic salmon MHC class II resulted in satisfactory binding, indicating that the proteins may represent candidates worthy of further exploration as potential vaccine candidates against pasteurellosis in salmon. Further work on antigen processing, MHC presentation and *in vivo* immunogenicity would be the next steps to corroborate these hypothetical computations.

Maltoporin-like proteins (Omp48) from several *Aeromonas* species have been used for vaccine development. Work by (Khushiramani et al. 2012) showed that recombinant Omp48 from *A. hydrophila* is immunogenic to fish where intramuscular vaccination of rohu (*L. rohita*) using the recombinant protein resulted in high titers of Omp46-specific antibodies. Upon intramuscular challenge, an RPS of approximately 70% was achieved in vaccinated compared to non-vaccinated fish. (Vàzquez-Juárez et al. 2004) showed that pre-incubation of polyclonal antibodies raised against epitopes of the maltoporin-like protein Omp48 with *A. veronii* resulted in inhibited adhesion to HeLa cells, demonstrating a neutralizing nature of antibodies produced. They also showed that preincubation of the purified Omp48 with HeLa cells blocked *A. veronii* from adhering to host cells through competitive inhibition and also provided cross-protection from binding with other species of *Aeromonas*, including *A. hydrophila* and *A. caviae*.

Some studies have shown limited protection conferred upon vaccination with OmpP1 (alternative name to FadL) isolated from non-typable *H. influenzae* (Bolduc et al., 2000) and *Klebsiella pneumoniae* (Hou et al., 2025), and subsequent challenge, noting that the high variability of the protein needs to be taken into consideration to improve protectivity conferred by the vaccines developed.

### 4.3 Influence of temperature on gene expression and implication for the industry

Bacteria can exploit environmental factors, such as temperature, to adapt their virulence patterns, by altering their gene expression (Fernández et al., 2007; Lages et al., 2019; Billaud et al., 2022). *Ph. atlanticus* subsp. *atlanticus* is able to grow successfully across a wide range of temperatures, and in a similar pattern, express virulence genes. There generally appears to be a positive correlation between temperature and gene expression, for both Omp47 and maltoporin. Our results show that for maltoporin, this becomes significant only above 20°C, while for Omp47, it becomes significant from 20°C upwards. However, Omp47gene expression at the late exponential stage at 20°C compared to 30°C is not significantly different, possibly indicating that a threshold level is reached for this virulence gene. Similar patterns of a correlation between temperature and increased virulence gene expression has been observed in other pathogenic bacteria. In *Vibrio parahaemolyticus*, adhesion and biofilm gene expression was increased at 31°C compared to 27°C, and the bacteria seemed to enter a VBNC state at 21°C (Billaud et al., 2022).

The increased expression of maltoporin and Omp47 at temperatures above 20°C may possibly translate to negative implications for thermal delousing processes in the Norwegian aquaculture industry. Currently, several procedures are employed by the industry, combining various techniques including freshwater and mechanical delousing with thermal methods. The temperatures reported as used for thermal delousing in such combinations vary but typically fall in the 28–34°C range (Bjørklund and Gismervik, 2025). 34°C is the maximum temperature currently allowed by the Norwegian Food Safety Authority for thermal delousing (Mattilsynet, 2019). As fish may be carriers and thus shedders of pathogenic and opportunistic bacteria, we hypothesize that the conditions present in well boats might thus be ideal for *Ph. atlanticus* subsp. *atlanticus* to replicate with increased virulence. This, coupled with the immunosuppressed condition of the fish due to the stressful nature of the delousing treatment, could lead to higher infection possibilities. It also could provide an explanation for the situations where outbreaks of pasteurellosis (and other bacterial diseases) are often observed within a short period of time following treatments (Stige et al., 2025). Although the treatment itself lasts less than a minute, the same water is used to treat all salmon present in that farm, a procedure which typically lasts hours. While this period in itself may not be long enough for the bacteria to grow to levels observed under controlled conditions, the myriad confounding factors mentioned above may greatly compound the severity of the situation. This raises a concern for fish welfare under non-medicinal delousing treatments.

Despite outbreaks of pasteurellosis in Atlantic salmon being more frequent in winter months compared to summer months, which seems to contradict the findings of this study, thermal delousing is carried out throughout the year. Given the sharp temperature gradient of the treatment chamber and the outside environment, the bacteria could still exploit the improved conditions for growth, and the increased immunosuppression of the host to colonize it. The implication here would be that the greater factor is increased stress of the host, which warrants further investigation to determine the connection to altered gene expression.

Initially, most outbreaks of *Ph. atlanticus* subsp. *atlanticus* since 2018 were confined to the Vestland region of the Norwegian coast (Nilsen, 2020). However, data from the Norwegian Veterinary Institute has shown that in 2025 outbreaks were detected from production area 1 up to production area 7 (Nilsen et al., 2026). This rapid spread of the species over such a short period of time could be attributed to horizontal spreading due to limited biosecurity protocols on well boats used for fish transport or non-medicinal delousing, or to gradually increasing sea temperatures, which favor the temperature tolerance of the bacteria.

### 4.4 Bacterial reservoirs and biosecurity

The reservoir for *Ph. atlanticus* subsp. *atlanticus* is still unknown, although genetic material from related *Phocoenobacter* species, including *Ph. uteri* have been isolated from both healthy and diseased marine mammals (Foster et al., 2000; Apprill et al., 2011; Godoy-Vitorino et al., 2017; Soares-Castro et al., 2019; Nilsen et al., 2026). It is also speculated that the transient nature of salmon pasteurellosis episodes recorded prior to 2017 could be attributed to spillover from unknown reservoirs which are thus epidemiological dead ends (Gulla et al., 2023). The connection has not yet been made as to when and how the spillover to a cold-blooded host occurred, either. However, the results of our work showing bacterial growth and upregulated gene expression at 30°C supports current research indicating potential ties to an as yet unknown marine mammal reservoir (Gulla et al., 2023).

Anecdotal evidence from surveys carried out by the Norwegian Veterinary Institute indicates that compared to autogenous vaccines, increased biosecurity on farms has led to similar if not better survival rates of salmon (Nilsen et al., 2025a). In work carried out by the Norwegian Veterinary Institute, it was found that survival of *Ph. atlanticus* subsp. *atlanticus* does not exceed 1 h in freshwater (Stige et al., 2025), which is promising for non-medicinal delousing treatments where combination methods of thermal and freshwater delousing are employed. This ties into growing evidence pointing toward the less virulent nature of *Ph. atlanticus* subsp. *atlanticus* compared to *Ph. atlanticus* subsp. *cyclopteri*.

### 4.5 Avenues for further research

The purpose of this study was to gain preliminary insight into the virulence mechanisms of *Ph. atlanticus* subsp. *atlanticus* and provide a platform for building on the results obtained, thus enhancing our understanding of the disease. It is important to stress the predictive nature of the current work. Expanding bioinformatic analysis to account for publicly available genomes of *Ph. atlanticus* subsp. *atlanticus* will help determine whether the results from this study reflect a population level situation. Functional analyses considering a wider variety of virulence factors aside from adhesins will increase the repertoire of potential immunogens available for vaccine development. The next steps will also include investigation of the impact of host-pathogen interactions on virulence gene expression, to assess the robustness and validity of *in silico* and *in vitro* studies of *Ph. atlanticus* subsp. *atlanticus* as well as *in vivo* investigations focusing on the immunogenicity of the pathogen and the related immune responses in Atlantic salmon.

## 5 Conclusion

Despite the significance of *Ph. atlanticus* subsp. *atlanticus* for causing pasteurellosis, particularly in salmon, little is known about its virulence factors. Identifying virulence factors in bacteria, and their expression profiles under different conditions, is essential for drug and vaccine development and for understanding courses of infections and how infections can be controlled or prevented. In this study we used a comprehensive bioinformatics pipeline to identify potential virulence factors in *Ph. atlanticus* subsp. *atlanticus*. This, together with our results from gene expression profiles, pave the way for future studies of the disease aiming at understanding mechanisms of infections involving *Ph. atlanticus* subsp. *atlanticus* and reducing outbreaks of pasteurellosis in aquaculture.

## Data Availability

The datasets presented in this study can be found in online repositories. This Whole Genome Shotgun project has been deposited at DDBJ/ENA/GenBank under the accession JBTTGU000000000. The version described in this paper is version JBTTGU010000000.
