# Correction: Virulence factors of *Phocoenobacter atlanticus* subspecies *atlanticus*: in search of vaccine targets

**DOI:** 10.3389/fmicb.2026.1897990

**Published:** 2026-06-16

**Authors:** Rebecca Marie Ellul, Hara Tselepidaki, Håkon Dahle, Håvard Skaar, Cyril Frantzen, Gyri Teien Haugland, Anita Rønneseth

**Affiliations:** 1 Department of Biological Sciences, University of Bergen, Bergen, Norway; 2 Institute of Marine Biology, Biotechnology and Aquaculture (IMBBC), Hellenic Center for Marine Research (HCMR), Heraklion, Greece; 3 STIM/ACD Pharma AS, Oslo, Norway

**Keywords:** Atlantic salmon, pasteurellosis, pathogenicity, *Phocoenobacter atlanticus* subsp. *atlanticus*, vaccine, virulence factors

The **Data availability statement** was erroneously given as “The datasets presented in this study can be found in online repositories. The names of the repository/repositories and accession number(s) can be found below https://www.ncbi.nlm.nih.gov/genbank/, PRJNA1403781.”

The correct **Data availability statement** is “The datasets presented in this study can be found in online repositories. This Whole Genome Shotgun project has been deposited at DDBJ/ENA/GenBank under the accession JBTTGU000000000. The version described in this paper is version JBTTGU010000000.”

In the published article, there was a mistake in section 2 **Materials and methods**, subsection 2.2 “*Genomic sequencing, assembly, and annotation*,” in the last sentence, which read: “This whole genome project has been deposited at GenBank under the Accession Number PRJNA1403781.”

The correct sentence is “This whole genome project has been deposited at GenBank under the Accession Number JBTTGU000000000.”

The original version of this article has been updated.

