# Supplementary material for: Virulence factors of Phocoenobacter atlanticus subspecies atlanticus: in search of vaccine targets
Source: Front Microbiol. 2026 May 18;17:1793695. doi: 10.3389/fmicb.2026.1793695 (PMC13224341; doi:10.3389/fmicb.2026.1793695)
Supplement: Supplementary file 1 [file Supplementary_file_1.pdf]

Supplementary Material

1 Supplementary Data

Supplementary Data 1: Two way ANOVA report for Maltoporin

Two Way Analysis of Variance 07 April 2026 19:53:41

Data source: Maltoporin in Growth curve analysisAPRIL2026.JNB Balanced Design
Dependent Variable: Fold change

Normality Test (Shapiro-Wilk): Passed (P = 0.994)

Equal Variance Test (Brown-Forsythe): Passed (P = 0.648)

Source of Variation	DF	SS	MS	F	P
Phase	2	4.638	2.319	10.778	<0.001
Temperature	2	18.403	9.201	42.763	<0.001
Phase x Temperature	4	9.488	2.372	11.023	<0.001
Residual	18	3.873	0.215		
Total	26	36.401	1.400		

Main effects cannot be properly interpreted if significant interaction is determined. This is because the size of a factor's effect depends upon the level of the other factor.

The effect of different levels of Phase depends on what level of Temperature is present. There is a statistically significant interaction between Phase and Temperature. (P = <0.001)

Power of performed test with alpha = 0.0500: for Phase : 0.961
Power of performed test with alpha = 0.0500: for Temperature : 1.000
Power of performed test with alpha = 0.0500: for Phase x Temperature : 0.997

Least square means for Phase :

Group	Mean
early	1.000
mid	1.677
late	1.994

Std Err of LS Mean = 0.155

Least square means for Temperature :

Group	Mean
15C	0.940
20C	1.007
30C	2.724

Std Err of LS Mean = 0.155

Least square means for Phase x Temperature :

Group	Mean
early x 15C	1.000
early x 20C	1.000
early x 30C	1.000
mid x 15C	0.573
mid x 20C	0.950
mid x 30C	3.508
late x 15C	1.246
late x 20C	1.072
late x 30C	3.663

Std Err of LS Mean = 0.268

All Pairwise Multiple Comparison Procedures (Holm-Sidak method):
Overall significance level = 0.05

Comparisons for factor: **Phase**

Comparison	Diff of Means	t	P	P<0.050
late vs. early	0.994	4.544	<0.001	Yes
mid vs. early	0.677	3.095	0.012	Yes
late vs. mid	0.317	1.449	0.165	No

Comparisons for factor: **Temperature**

Comparison	Diff of Means	t	P	P<0.050
30C vs. 15C	1.784	8.160	<0.001	Yes
30C vs. 20C	1.716	7.850	<0.001	Yes
20C vs. 15C	0.0678	0.310	0.760	No

Comparisons for factor: **Temperature within early**

Comparison	Diff of Means	t	P	P<0.050
15C vs. 30C	6.661E-16	1.759E-15	1.000	No
15C vs. 20C	6.661E-16	1.759E-15	1.000	No
20C vs. 30C	0.000	0.000	1.000	No

Comparisons for factor: **Temperature within mid**

Comparison	Diff of Means	t	P	P<0.050
30C vs. 15C	2.935	7.750	<0.001	Yes
30C vs. 20C	2.558	6.755	<0.001	Yes
20C vs. 15C	0.377	0.995	0.333	No

Comparisons for factor: **Temperature within late**

Comparison	Diff of Means	t	P	P<0.050
30C vs. 20C	2.591	6.841	<0.001	Yes
30C vs. 15C	2.417	6.383	<0.001	Yes
15C vs. 20C	0.174	0.459	0.652	No

Comparisons for factor: **Phase within 15C**

Comparison	Diff of Means	t	P	P<0.050
late vs. mid	0.673	1.777	0.252	No
early vs. mid	0.427	1.128	0.473	No
late vs. early	0.246	0.649	0.525	No

Comparisons for factor: **Phase within 20C**

Comparison	Diff of Means	t	P	P<0.050
late vs. mid	0.122	0.323	0.984	No
late vs. early	0.0722	0.191	0.984	No
early vs. mid	0.0502	0.133	0.984	No

Comparisons for factor: **Phase within 30C**

Comparison	Diff of Means	t	P	P<0.050
late vs. early	2.663	7.032	<0.001	Yes
mid vs. early	2.508	6.622	<0.001	Yes
late vs. mid	0.155	0.410	0.687	No

Supplementary Data 2: Two way ANOVA report for Omp47

Two Way Analysis of Variance 07 April 2026 18:46:29

Data source: Omp47 in Growth curve analysisAPRIL2026.JNB

Balanced Design

Dependent Variable: Fold change

Normality Test (Shapiro-Wilk): Passed (P = 0.177)

Equal Variance Test (Brown-Forsythe): Passed (P = 0.694)

Source of Variation	DF	SS	MS	F	P
Phase	2	3.912	1.956	23.202	<0.001
Temperature	2	5.710	2.855	33.870	<0.001
Phase x Temperature	4	3.590	0.897	10.646	<0.001
Residual	18	1.517	0.0843		
Total	26	14.729	0.566		

Main effects cannot be properly interpreted if significant interaction is determined. This is because the size of a factor's effect depends upon the level of the other factor.

The effect of different levels of Phase depends on what level of Temperature is present. There is a statistically significant interaction between Phase and Temperature. (P = <0.001)

Power of performed test with alpha = 0.0500: for Phase : 1.000
Power of performed test with alpha = 0.0500: for Temperature : 1.000
Power of performed test with alpha = 0.0500: for Phase x Temperature : 0.997

Least square means for Phase :

**Group**	**Mean**
early	1.000
mid	1.437
late	1.932

Std Err of LS Mean = 0.0968

Least square means for Temperature :

**Group**	**Mean**
15C	0.877
20C	1.490
30C	2.002

Std Err of LS Mean = 0.0968
Least square means for Phase x Temperature :

**Group**	**Mean**
early x 15C	1.000
early x 20C	1.000
early x 30C	1.000
mid x 15C	0.454
mid x 20C	1.318
mid x 30C	2.540
late x 15C	1.178
late x 20C	2.151
late x 30C	2.467

Std Err of LS Mean = 0.168

All Pairwise Multiple Comparison Procedures (Holm-Sidak method): Overall significance level = 0.05

Comparisons for factor: **Phase**

**Comparison**	**Diff of Means**	**t**	**P**	**P<0.050**
late vs. early	0.932	6.808	<0.001	Yes
late vs. mid	0.494	3.613	0.004	Yes
mid vs. early	0.437	3.195	0.005	Yes

Comparisons for factor: **Temperature**

**Comparison**	**Diff of Means**	**t**	**P**	**P<0.050**
30C vs. 15C	1.125	8.220	<0.001	Yes
20C vs. 15C	0.612	4.474	<0.001	Yes
30C vs. 20C	0.513	3.746	0.001	Yes

Comparisons for factor: **Temperature within early**

**Comparison**	**Diff of Means**	**t**	**P**	**P<0.050**
20C vs. 15C	2.220E-15	9.367E-15	1.000	No
20C vs. 30C	6.661E-16	2.810E-15	1.000	No
30C vs. 15C	1.554E-15	6.557E-15	1.000	No

Comparisons for factor: **Temperature within mid**

**Comparison**	**Diff of Means**	**t**	**P**	**P<0.050**
30C vs. 15C	2.086	8.800	<0.001	Yes
30C vs. 20C	1.222	5.156	<0.001	Yes
20C vs. 15C	0.864	3.643	0.002	Yes

Comparisons for factor: **Temperature within late**

**Comparison**	**Diff of Means**	**t**	**P**	**P<0.050**
30C vs. 15C	1.289	5.437	<0.001	Yes
20C vs. 15C	0.973	4.105	0.001	Yes
30C vs. 20C	0.316	1.332	0.200	No

Comparisons for factor: **Phase within 15C**

**Comparison**	**Diff of Means**	**t**	**P**	**P<0.050**
late vs. mid	0.724	3.052	0.020	Yes
early vs. mid	0.546	2.303	0.066	No
late vs. early	0.178	0.749	0.463	No

Comparisons for factor: **Phase within 20C**

**Comparison**	**Diff of Means**	**t**	**P**	**P<0.050**
late vs. early	1.151	4.855	<0.001	Yes
late vs. mid	0.833	3.515	0.005	Yes
mid vs. early	0.318	1.340	0.197	No

Comparisons for factor: **Phase within 30C**

**Comparison**	**Diff of Means**	**t**	**P**	**P<0.050**
mid vs. early	1.540	6.497	<0.001	Yes

late vs. early	1.467	6.187	<0.001	Yes
mid vs. late	0.0734	0.310	0.760	No

2 Supplementary Figures and Tables

2.1 Supplementary Figure

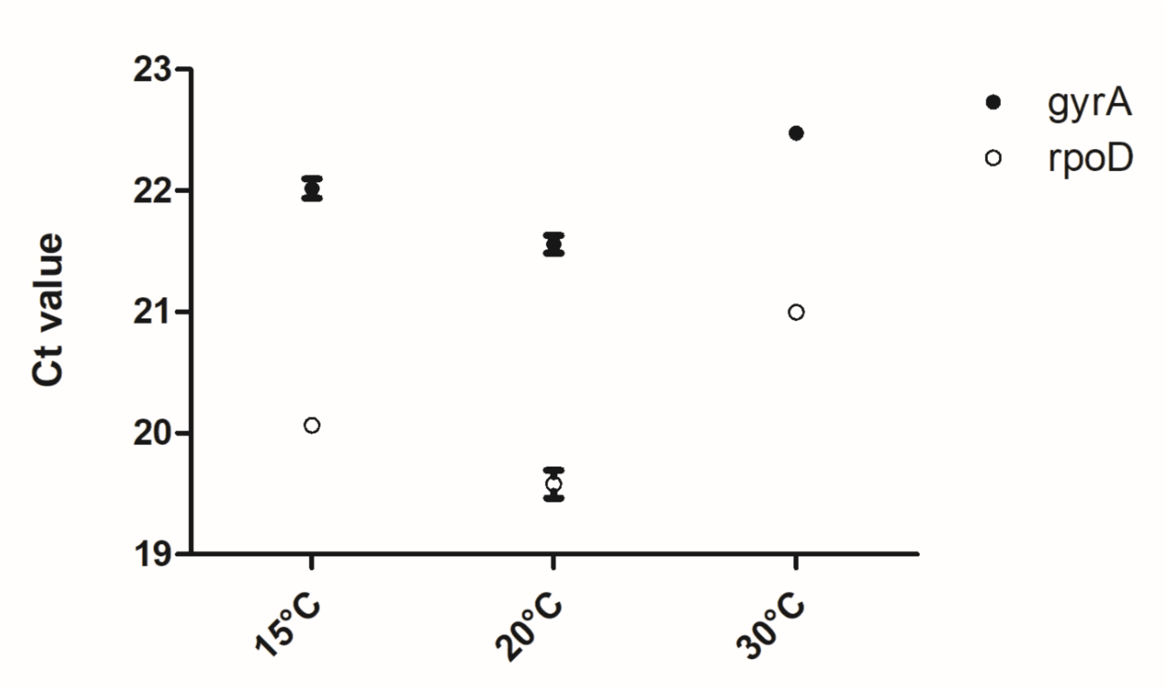

Supplementary Figure 1. Ct values of the two reference genes across the three temperatures tested. Error bars show standard deviation.

2.2 Supplementary Tables

Supplementary Table 1:

Percent identity of maltoporin from Paa-1-UiB-2019 compared to publicly available *Ph. atlanticus* genomes. Based on data from Gulla *et al*. 2023.

Species	Code	Percent identity	Host	Year	Region	Group
Ph. atlanticus subsp. *atlanticus*	VIB3834	100	Salmon	2020	Vestland	Pas4

Ph. atlanticus subsp. *atlanticus*	VIB3791		Salmon	2019	Vestland	Pas4
Ph. atlanticus subsp. *atlanticus*	VIB3790		Salmon	2019	Vestland	Pas4
Ph. atlanticus subsp. *atlanticus*	VIB3789		Salmon	2019	Vestland	Pas4
Ph. atlanticus subsp. *atlanticus*	VIB3788		Salmon	2019	Vestland	Pas4
Ph. atlanticus subsp. *atlanticus*	VIB3787		Salmon	2019	Vestland	Pas4
Ph. atlanticus subsp. *atlanticus*	VIB3786		Salmon	2019	Vestland	Pas4
Ph. atlanticus subsp. *atlanticus*	VIB3785		Salmon	2019	Vestland	Pas4
Ph. atlanticus subsp. *atlanticus*	VIB3784		Salmon	2019	Vestland	Pas4
Ph. atlanticus subsp. *atlanticus*	VIB3783		Salmon	2019	Vestland	Pas4
Ph. atlanticus subsp. *atlanticus*	VIB3782		Salmon	2019	Rogaland	Pas4
Ph. atlanticus subsp. *atlanticus*	VIB3781		Salmon	2018	Rogaland	Pas4
Ph. atlanticus subsp. *atlanticus*	VIB3770		Salmon	2020	Vestland	Pas4
Ph. atlanticus subsp. *atlanticus*	VIB3767		Salmon	2020	Vestland	Pas4
Ph. atlanticus subsp. *atlanticus*	VIB3766		Salmon	2020	Vestland	Pas4

Ph. atlanticus subsp. *atlanticus*	VIB3764		Salmon	2020	Vestland	Pas4
Ph. atlanticus subsp. *atlanticus*	NVIB3763		Salmon	2020	Vestland	Pas4
Ph. atlanticus subsp. *atlanticus*	VIB3760		Salmon	2020	Vestland	Pas4
Ph. atlanticus subsp. *atlanticus*	VIB3711		Salmon	2020	Vestland	Pas4
Ph. atlanticus subsp. *atlanticus*	VIB3710		Salmon	2020	Vestland	Pas4
Ph. atlanticus subsp. *atlanticus*	VIB3709		Salmon	2020	Vestland	Pas4
Ph. atlanticus subsp. *atlanticus*	NVIB3708		Salmon	2020	Vestland	Pas4
Ph. atlanticus subsp. *atlanticus*	VIB3707		Salmon	2020	Vestland	Pas4
Ph. atlanticus subsp. *atlanticus*	NVIB3694		Salmon	2012	Vestland	Pas3
Ph. atlanticus subsp. *atlanticus*	NVIB3687		Salmon	2019	MøreRomsdal	Pas4
Ph. atlanticus subsp. *atlanticus*	VIB3673		Salmon	2019	Vestland	Pas4
Ph. atlanticus subsp. *atlanticus*	NVIB3672		Salmon	2019	Vestland	Pas4
Ph. atlanticus subsp. *atlanticus*	VIB3670		Salmon	2019	Vestland	Pas4
Ph. atlanticus subsp. *atlanticus*	VIB3649		Salmon	2019	Vestland	Pas4

Ph. atlanticus subsp. *atlanticus*	NVIB3642		Salmon	2019	Vestland	Pas4
Ph. atlanticus subsp. *atlanticus*	NVIB3624		Salmon	2019	Vestland	Pas4
Ph. atlanticus subsp. *atlanticus*	VIB3528		Salmon	2019	MøreRomsdal	Pas4
Ph. atlanticus subsp. *atlanticus*	VIB3506		Salmon	2019	Vestland	Pas4
Ph. atlanticus subsp. *atlanticus*	VIB3490		Lumpfish	2019	Vestland	Pas4
Ph. atlanticus subsp. *atlanticus*	VIB3309		Salmon	2019	Vestland	Pas4
Ph. atlanticus subsp. *atlanticus*	VIB3308		Salmon	2019	Vestland	Pas4
Ph. atlanticus subsp. *atlanticus*	VIB3305		Salmon	2019	Vestland	Pas4
Ph. atlanticus subsp. *atlanticus*	VIB3296		Salmon	2019	Vestland	Pas4
Ph. atlanticus subsp. *atlanticus*	VIB3289		Salmon	2019	Vestland	Pas4
Ph. atlanticus subsp. *atlanticus*	VIB3243		Salmon	2019	Vestland	Pas4
Ph. atlanticus subsp. *atlanticus*	VIB3014		Salmon	2018	Vestland	Pas4
Ph. atlanticus subsp. *atlanticus*	VIB2955		Salmon	2018	Rogaland	Pas4
Ph. atlanticus subsp. *atlanticus*	VIB2921		Salmon	2018	Rogaland	Pas4

Ph. atlanticus subsp. *atlanticus*	VIB2913		Salmon	2018	Rogaland	Pas4
Ph. atlanticus subsp. *atlanticus*	VIB2829		Salmon	2018	Vestland	Pas4
Ph. atlanticus subsp. *atlanticus*	VIB2717		Lumpfish	2018	Vestland	Pas4
Ph. atlanticus subsp. *atlanticus*	VIB2680		Salmon	2018	Vestland	Pas4
Ph. atlanticus subsp. *atlanticus*	NVIB2635		Salmon	2018	Vestland	Pas4
Ph. atlanticus subsp. *atlanticus*	VIB3695		Salmon	2000	Vestland	Pas2
Ph. atlanticus subsp. *atlanticus*	VIB3693	82.38	Salmon	1999	Vestland	Pas2
Ph. atlanticus subsp. *atlanticus*	NVIB3131		Salmon	1999	Vestland	Pas2
Ph. atlanticus subsp. *cyclopteri*	NVIO 9100		Lumpfish	2013	MøreRomsdal	Pac
Ph. atlanticus subsp. *cyclopteri*	NVI-9258		Lumpfish	2013	Vestland	Pac
Ph. atlanticus subsp. *cyclopteri*	NVIO3648		Lumpfish	1996	Troms	Pac
Ph. atlanticus subsp. *cyclopteri*	NVIB543	79.65	Lumpfish	2013	Rogaland	Pac
Ph. atlanticus subsp. *cyclopteri*	VIB3804		Lumpfish	2013	Nordland	Pac
Ph. atlanticus subsp. *cyclopteri*	NVIB3802		Lumpfish	2013	Nordland	Pac

Ph. atlanticus subsp. *cyclopteri*	NVIB3801		Lumpfish	2017	Troms	Pac
Ph. atlanticus subsp. *cyclopteri*	VIB3703		Lumpfish	2020	Vestland	Pac
Ph. atlanticus subsp. *cyclopteri*	VIB3468		Lumpfish	2019	Vestland	Pac
Ph. atlanticus subsp. *cyclopteri*	VIB3273		Lumpfish	2019	Rogaland	Pac
Ph. atlanticus subsp. *cyclopteri*	VIB3004		Lumpfish	2018	Vestland	Pac
Ph. atlanticus subsp. *cyclopteri*	NVIB2993		Lumpfish	2018	Vestland	Pac
Ph. atlanticus subsp. *cyclopteri*	NVIB2702		Lumpfish	2018	Vestland	Pac
Ph. atlanticus subsp. *cyclopteri*	NVIB1926		Lumpfish	2017	Vestland	Pac
Ph. atlanticus subsp. *cyclopteri*	NVIB164		Lumpfish	2012	Rogaland	Pac
Ph. atlanticus subsp. *cyclopteri*	VIB1366		Lumpfish	2016	Vestland	Pac
Ph. atlanticus subsp. *cyclopteri*	NVIB1365		Lumpfish	2016	Vestland	Pac
Ph. atlanticus subsp. *cyclopteri*	VIB1234		Lumpfish	2015	Vestland	Pac
Ph. atlanticus subsp. *cyclopteri*	TW141_17		Lumpfish	2017	Scotland	Pac
Ph. atlanticus subsp. *cyclopteri*	TW138_17		Lumpfish	2017	Scotland	Pac

Ph. atlanticus subsp. *atlanticus*	VIB3692	78.25	Salmon	1992	Troms	Pas1
Ph. atlanticus subsp. *atlanticus*	VIB3691		Salmon	1992	Troms	Pas1
Ph. atlanticus subsp. *atlanticus*	NVIB3689		Salmon	1991	Troms	Pas1

Supplementary Table 2:

Percent identity of Omp47 from Paa-1-UiB-2019 compared to publicly available *Ph. atlanticus* genomes. Based on data from Gulla *et al.* 2023.

**Species**	**Code**	**Percent identity**	**Host**	**Year**	**Region**	**Code**
Ph. atlanticus subsp. *atlanticus*	VIB3692	99.76	Salmon	1992	Troms	Pas1
Ph. atlanticus subsp. *atlanticus*	VIB3691		Salmon	1992	Troms	Pas1
Ph. atlanticus subsp. *atlanticus*	NVIB3689		Salmon	1991	Troms	Pas1
Ph. atlanticus subsp. *atlanticus*	VIB3834		Salmon	2020	Vestland	Pas4
Ph. atlanticus subsp. *atlanticus*	VIB3791		Salmon	2019	Vestland	Pas4
Ph. atlanticus subsp. *atlanticus*	VIB3790		Salmon	2019	Vestland	Pas4
Ph. atlanticus subsp. *atlanticus*	VIB3789		Salmon	2019	Vestland	Pas4
Ph. atlanticus subsp. *atlanticus*	VIB3788		Salmon	2019	Vestland	Pas4

Ph. atlanticus subsp. *atlanticus*	VIB3787		Salmon	2019	Vestland	Pas4
Ph. atlanticus subsp. *atlanticus*	VIB3786		Salmon	2019	Vestland	Pas4
Ph. atlanticus subsp. *atlanticus*	VIB3785		Salmon	2019	Vestland	Pas4
Ph. atlanticus subsp. *atlanticus*	VIB3784		Salmon	2019	Vestland	Pas4
Ph. atlanticus subsp. *atlanticus*	VIB3783		Salmon	2019	Vestland	Pas4
Ph. atlanticus subsp. *atlanticus*	VIB3782		Salmon	2019	Rogaland	Pas4
Ph. atlanticus subsp. *atlanticus*	VIB3781		Salmon	2018	Rogaland	Pas4
Ph. atlanticus subsp. *atlanticus*	VIB3770		Salmon	2020	Vestland	Pas4
Ph. atlanticus subsp. *atlanticus*	VIB3767		Salmon	2020	Vestland	Pas4
Ph. atlanticus subsp. *atlanticus*	VIB3766		Salmon	2020	Vestland	Pas4
Ph. atlanticus subsp. *atlanticus*	VIB3764		Salmon	2020	Vestland	Pas4
Ph. atlanticus subsp. *atlanticus*	NVIB3763		Salmon	2020	Vestland	Pas4
Ph. atlanticus subsp. *atlanticus*	VIB3760		Salmon	2020	Vestland	Pas4
Ph. atlanticus subsp. *atlanticus*	VIB3711		Salmon	2020	Vestland	Pas4

Ph. atlanticus subsp. *atlanticus*	VIB3710		Salmon	2020	Vestland	Pas4
Ph. atlanticus subsp. *atlanticus*	VIB3709		Salmon	2020	Vestland	Pas4
Ph. atlanticus subsp. *atlanticus*	NVIB3708		Salmon	2020	Vestland	Pas4
Ph. atlanticus subsp. *atlanticus*	VIB3707		Salmon	2020	Vestland	Pas4
Ph. atlanticus subsp. *atlanticus*	VIB3695		Salmon	2000	Vestland	Pas2
Ph. atlanticus subsp. *atlanticus*	NVIB3694		Salmon	2012	Vestland	Pas3
Ph. atlanticus subsp. *atlanticus*	VIB3693		Salmon	1999	Vestland	Pas2
Ph. atlanticus subsp. *atlanticus*	NVIB3687		Salmon	2019	MøreRomsdal	Pas4
Ph. atlanticus subsp. *atlanticus*	VIB3673		Salmon	2019	Vestland	Pas4
Ph. atlanticus subsp. *atlanticus*	NVIB3672		Salmon	2019	Vestland	Pas4
Ph. atlanticus subsp. *atlanticus*	VIB3670		Salmon	2019	Vestland	Pas4
Ph. atlanticus subsp. *atlanticus*	VIB3649		Salmon	2019	Vestland	Pas4
Ph. atlanticus subsp. *atlanticus*	NVIB3642		Salmon	2019	Vestland	Pas4
Ph. atlanticus subsp. *atlanticus*	NVIB3624		Salmon	2019	Vestland	Pas4

Ph. atlanticus subsp. *atlanticus*	VIB3528		Salmon	2019	MøreRomsdal	Pas4
Ph. atlanticus subsp. *atlanticus*	VIB3506		Salmon	2019	Vestland	Pas4
Ph. atlanticus subsp. *atlanticus*	VIB3490		Lumpfish	2019	Vestland	Pas4
Ph. atlanticus subsp. *atlanticus*	VIB3309		Salmon	2019	Vestland	Pas4
Ph. atlanticus subsp. *atlanticus*	VIB3308		Salmon	2019	Vestland	Pas4
Ph. atlanticus subsp. *atlanticus*	VIB3305		Salmon	2019	Vestland	Pas4
Ph. atlanticus subsp. *atlanticus*	VIB3296		Salmon	2019	Vestland	Pas4
Ph. atlanticus subsp. *atlanticus*	VIB3289		Salmon	2019	Vestland	Pas4
Ph. atlanticus subsp. *atlanticus*	VIB3243		Salmon	2019	Vestland	Pas4
Ph. atlanticus subsp. *atlanticus*	NVIB3131		Salmon	1999	Vestland	Pas2
Ph. atlanticus subsp. *atlanticus*	VIB3014		Salmon	2018	Vestland	Pas4
Ph. atlanticus subsp. *atlanticus*	VIB2955		Salmon	2018	Rogaland	Pas4
Ph. atlanticus subsp. *atlanticus*	VIB2921		Salmon	2018	Rogaland	Pas4
Ph. atlanticus subsp. *atlanticus*	VIB2913		Salmon	2018	Rogaland	Pas4

Ph. atlanticus subsp. *atlanticus*	VIB2829		Salmon	2018	Vestland	Pas4
Ph. atlanticus subsp. *atlanticus*	VIB2717		Lumpfish	2018	Vestland	Pas4
Ph. atlanticus subsp. *atlanticus*	VIB2680		Salmon	2018	Vestland	Pas4
Ph. atlanticus subsp. *atlanticus*	NVIB2635		Salmon	2018	Vestland	Pas4
Ph. atlanticus subsp. *cyclopteri*	NVIO 9100	63.45	Lumpfish	2013	MøreRomsdal	Pac
Ph. atlanticus subsp. *cyclopteri*	NVI-9258		Lumpfish	2013	Vestland	Pac
Ph. atlanticus subsp. *cyclopteri*	NVIO3648		Lumpfish	1996	Troms	Pac
Ph. atlanticus subsp. *cyclopteri*	NVIB543		Lumpfish	2013	Rogaland	Pac
Ph. atlanticus subsp. *cyclopteri*	VIB3804		Lumpfish	2013	Nordland	Pac
Ph. atlanticus subsp. *cyclopteri*	NVIB3802		Lumpfish	2013	Nordland	Pac
Ph. atlanticus subsp. *cyclopteri*	NVIB3801		Lumpfish	2017	Troms	Pac
Ph. atlanticus subsp. *cyclopteri*	VIB3703		Lumpfish	2020	Vestland	Pac
Ph. atlanticus subsp. *cyclopteri*	VIB3468		Lumpfish	2019	Vestland	Pac
Ph. atlanticus subsp. *cyclopteri*	VIB3273		Lumpfish	2019	Rogaland	Pac

Ph. atlanticus subsp. *cyclopteri*	VIB3004		Lumpfish	2018	Vestland	Pac
Ph. atlanticus subsp. *cyclopteri*	NVIB2993		Lumpfish	2018	Vestland	Pac
Ph. atlanticus subsp. *cyclopteri*	NVIB2702		Lumpfish	2018	Vestland	Pac
Ph. atlanticus subsp. *cyclopteri*	NVIB1926		Lumpfish	2017	Vestland	Pac
Ph. atlanticus subsp. *cyclopteri*	NVIB164		Lumpfish	2012	Rogaland	Pac
Ph. atlanticus subsp. *cyclopteri*	VIB1366		Lumpfish	2016	Vestland	Pac
Ph. atlanticus subsp. *cyclopteri*	NVIB1365		Lumpfish	2016	Vestland	Pac
Ph. atlanticus subsp. *cyclopteri*	VIB1234		Lumpfish	2015	Vestland	Pac
Ph. atlanticus subsp. *cyclopteri*	TW141_17		Lumpfish	2017	Scotland	Pac
Ph. atlanticus subsp. *cyclopteri*	TW138_17		Lumpfish	2017	Scotland	Pac

Supplementary Table 3:

P values for comparisons of gene expression across temperatures.

	**Maltoporin**			**Omp47**		
	**Early**	**Mid**	**Late**	**Early**	**Mid**	**Late**
**15°C v. 20°C**	1.000	0.333	0.652	1.000	0.002	<0.001

**15°C v. 30°C**	1.000	<0.001	<0.001	1.000	<0.001	<0.001
**20°C v. 30°C**	1.000	<0.001	<0.001	1.000	<0.001	0.200
